# Editorial: Advances in therapeutic gastrointestinal endoscopy: from bench to bedside

**DOI:** 10.3389/fmed.2025.1647463

**Published:** 2025-07-04

**Authors:** Jia Xu, Xiaowei Tang

**Affiliations:** Department of Gastroenterology, The Affiliated Hospital of Southwest Medical University, Luzhou, China

**Keywords:** therapeutic gastrointestinal endoscopy, artificial intelligence, innovative endoscopic techniques, endoscopic ultrasound, evolution

Gastrointestinal (GI) diseases such as cancers, inflammatory bowel disease, and functional GI disorders pose significant global health challenges, emphasizing the critical need for effective diagnostic and therapeutic interventions (1). Over recent decades, therapeutic gastrointestinal endoscopy has transitioned remarkably from a diagnostic adjunct to a primary treatment modality, incorporating advanced techniques such as endoscopic mucosal resection (EMR), submucosal dissection (ESD), and peroral endoscopic myotomy (POEM) (2, 3). These advances, rooted in foundational biomedical research, imaging innovations, and emerging artificial intelligence applications, have dramatically improved procedural outcomes and patient safety. However, bridging fundamental research breakthroughs with clinical practice remains a dynamic and essential pursuit. This Research Topic highlights recent multidisciplinary efforts in this field, showcasing translational studies that directly enhance clinical effectiveness and patient care, while identifying future directions to further refine therapeutic endoscopic strategies.

Recent advancements in artificial intelligence (AI) integration with endoscopic imaging have demonstrated promising outcomes. A notable recent study has explored the combined use of white-light imaging (WLI) and narrow-band imaging (NBI)-based AI models to enhance the precision of endoscopic remission assessment (4). This study highlights that the integration of both imaging techniques significantly improved specificity from 42.2% for WLI alone to 61.5% for the combined approach, without compromising sensitivity. The dual-model AI approach facilitates better risk stratification, offering clinicians a more reliable and personalized strategy to predict relapse and manage ulcerative colitis proactively. This development underscores the potential of multidimensional AI-enhanced imaging modalities as practical tools for bridging foundational biomedical innovations and direct clinical applications, emphasizing the continued need for multidisciplinary translational research to optimize patient care outcomes.

Innovative endoscopic techniques continue to evolve, offering enhanced procedural efficacy and patient safety. For instance, a recent advancement utilizes a novel method of clip and dental floss traction-assisted endoscopic mucosal resection, demonstrating improved success rates in managing challenging lesions such as early carcinoma of the duodenal papilla (5). Additionally, the development of customized devices like the adjustable snare-based extraction tool for giant gastric bezoars illustrates how innovative designs can overcome limitations of conventional equipment (6). These novel techniques underscore the significance of translational innovation, combining practical engineering solutions with clinical expertise to refine therapeutic gastrointestinal endoscopy, ultimately optimizing clinical outcomes.

In the realm of managing malignant gastric outlet obstruction (GOO), endoscopic ultrasound-guided gastroenterostomy (EUS-GE) has rapidly emerged as a first-line therapeutic option, rivaling traditional duodenal stenting. EUS-GE not only overcomes limitations such as stent obstruction but also demonstrates a comparable safety profile, as evidenced by multiple prospective studies and randomized controlled trials. A recent multicenter retrospective analysis compared two prevalent EUS-GE techniques—the direct technique over a guidewire (DTOG) and the wireless endoscopic simplified technique (WEST) (7). The findings revealed significantly higher technical success with WEST and a markedly lower adverse event rate (14.6% vs. 46.7%). The study highlighted the importance of target loop stabilization, a key procedural step, which WEST achieves through saline infusion via an oroenteric catheter, resulting in small bowel distension and improved alignment with the gastric wall for lumen-apposing metal stent (LAMS) deployment. As EUS-GE continues to evolve toward greater standardization and simplification, it offers a minimally invasive, effective alternative for high-risk or inoperable patients, further exemplifying the translational impact of advanced endoscopic innovations on clinical practice.

Looking forward, the trajectory of therapeutic gastrointestinal endoscopy continues to align closely with advances in precision medicine, biomedical engineering, and digital health. Future developments are expected to include real-time molecular endoscopic imaging for on-the-spot histological assessment, robotics-assisted navigation to enhance procedural dexterity in complex anatomies, and further integration of machine learning algorithms for intraoperative decision support. Moreover, scalable training platforms—potentially powered by AI-driven simulators and augmented reality—will be essential for ensuring consistent operator proficiency and expanding global access to advanced endoscopic care. Addressing current limitations such as device cost, procedural standardization, and equitable access will require sustained multidisciplinary collaboration among clinicians, engineers, data scientists, and policymakers. These future directions will further narrow the gap between technological innovation and clinical implementation.

In conclusion, the recent strides in therapeutic gastrointestinal endoscopy—from AI-enhanced diagnostics and novel resection tools to cutting-edge EUS-guided interventions—exemplify the field's dynamic evolution toward more precise, safe, and effective patient care. Each innovation represents a meaningful step in the ongoing journey from bench to bedside, where foundational discoveries are translated into tangible clinical impact. Continued investment in translational research, collaborative development, and clinical validation will be vital in driving this transformation forward. By sustaining this integrative momentum, the field is well-positioned to redefine standards of care and ultimately improve outcomes for patients with complex gastrointestinal diseases.
